# Investigation of the PD-1 and PD-L1 Immune Checkpoint Molecules Throughout Healthy Human Pregnancy and in Nonpregnant Women

**DOI:** 10.3390/jcm9082536

**Published:** 2020-08-06

**Authors:** Matyas Meggyes, David U. Nagy, Laszlo Szereday

**Affiliations:** 1 Department of Medical Microbiology and Immunology, Medical School, University of Pecs, 7624 Pecs, Hungary; szereday.laszlo@pte.hu; 2 Janos Szentagothai Research Centre, 7624 Pecs, Hungary; 3 Medical Centre, Cochrane Hungary, University of Pécs, 7623 Pécs, Hungary; davenagy9@gmail.com

**Keywords:** PD-1, PD-L1, pregnancy, immune checkpoint, NKG2D

## Abstract

**Background**: A growing body of evidence supports the importance of PD-1 and PD-L1, especially in the materno-fetal interface, although limited information is available about the peripheral expression of these molecules during the trimesters of pregnancy. **Methods**: 13 healthy women were enrolled from the 1st, 10 from the 2nd and 12 from the 3rd trimester of pregnancy at the same time, 10 healthy, age-matched nonpregnant women formed the control group. From peripheral blood, mononuclear cells were separated and stored at –80 °C. From freshly thawed samples, surface and intracellular staining were performed for flow cytometric analyses. CD107a degranulation assay was used to evaluate the cytotoxicity. **Results**: significant alternation was detected in PD-1 expression by CD8+T cells and in PD-L1 expression by CD8+T, CD4+T and Treg cells. An interesting relationship was revealed between the PD-1 and PD-L1 expression by the investigated subpopulations in 2nd trimester of pregnancy. Different expression patterns of an activation receptor NKG2D by the PD-1+ CD8+T cells was observed during pregnancy. The notable relationship was further determined in cytotoxicity between PD-1+ and NKG2D+ CD8+T cells throughout pregnancy. **Conclusions**: the different PD-1 presence and the relationship with NKG2D could contribute to the dynamic changes of the Th1 and Th2 predominance throughout the three trimesters of a healthy pregnancy.

## 1. Introduction

During a healthy pregnancy, the adaptation of the maternal immune system is mandatory to support adequate fetal development. Maternal immune tolerance is a complex reaction of the immune system to the presence of the fetus, which is much more complicated than previously thought. Thirty years ago, the theory of Th1/Th2 shift was usually used to explain the phenomenon of maternal immunotolerance since Th2-type immune responses are more dominant than the Th1-type ones, therefore protecting the fetus from maternal Th1-type immunity and providing proper fetal development [1]. In the 2000s, the Th1/Th2 thesis was completed into the Th1/Th2/Th17 and Regulatory T (Treg) cells paradigm [2], which added a more complex explanation about pregnancy-related immune regulation [3,4,5]. T-helper cells can be classified into Th1 cells, which produce interleukin IL-2, IL-10 IFN-γ and TNF-α/β and are involved in cellular immunity, Th2 cells, which produce IL-4, IL-5 and IL-13 and are involved in humoral immunity and Th17 cells, which produce the proinflammatory cytokine, IL-17, play important roles for the induction of inflammation. Now, it is established that during the 1st trimester, Th1 immunity is predominant, and this controlled inflammation is required for the implantation processes and early development of the embryo. Turning to the 2nd trimester, Th2 immune responses will be dominant hence promoting fetal allograft tolerance and evoking a balance between the mother and the fetus. At the end of the pregnancy, Th1 immunity will be dominant again and play a significant role in the induction of labor [6]. This alternation of the Th1 and Th2 prevalence of the immune responses during pregnancy is primarily restricted to the materno-fetal interface, although our knowledge about the periphery is unclear.

The full activation of T cells depends on two different signals: signal one is derived from the interaction between antigenic peptide Major Histocompatibility Complex (MHC) on the surface of Antigen Presenting Cells (APCs) and the T cell receptor, and signal two requires antigen-independent co-signaling molecules. T cell activation is tightly regulated by co-stimulators or co-inhibitors known as immune checkpoints.

These immune checkpoint molecules are the key players in maintaining immunological homeostasis. These receptor-ligand interactions are provided by a co-activatory or/and co-inhibitory signal, which are crucial in optional T cell activation. Co-stimulatory signals through activating receptors can induce T cell proliferation and cytotoxic functions. At the same time, co-inhibitory checkpoint molecules could downregulate the effector cells, reduce their cytotoxicity or induce apoptosis.

Programmed cell death-1 (PD-1, CD279) is a transmembrane receptor that encoded by the *PDCD1* gene [7] and a novel part of the B7− CD28 immunoglobulin family. PD-1 is one of the most investigated inhibitory receptors and primarily expressed on the surface of T-cells, B cells, monocytes, dendritic cells and Treg too [8,9]. Although under physiological conditions PD-1 expression can be constitutive on immature CD4− and CD8− thymocytes, activated CD4+ and CD8+T cells, B cells, monocytes, Natural Killer (NK) cells and Dendritic Cells (DCs) it can also be induced by different cytokines including IL-10 or TGF-b on APCs, myeloid DC and monocytes [10] as well as under different pathological conditions such as preeclampsia, chronic viral infections and cancer. Moreover, the high expression of PD-1 molecule is a characteristic of exhausted T cells [10,11]. On the other hand, PD-L1 expression which is constitutive in different host cells and immune cells including activated T and B cells, DCs and monocytes, can also be induced by different inflammatory cytokines and interferon INF-g [10,12], being highly expressed by different cancer cells. PD-1 mediated co-inhibitory signal is manifested after binding its ligand molecules PD-L1 (B7-H1; CD274) and PD-L2 (B7-DC; CD273). Both ligands are expressed on the surface of APCs, including macrophages and DCs while PD-L1 is present on resting T cells, B cells, endothelial- and epithelial cells [13,14]. Several tumor types also express PD-L1, including NSCLC, renal cell carcinoma, melanoma and gastric cancer [15,16,17]. The connection of PD-1 with PD-L1 results in an inhibition of the T cell activation hence can induce peripheral tolerance [18] or redound tumor immune escape mechanisms [19]. The PD-1/PD-L1 pathway plays an important role in the regulation of immune suppression via reduced T cell proliferation, induced T cell anergy and exhaustion, diminished cytokine production and increased Treg cell function [20].

Besides the extensive research in the field of tumor immunology in recent years, many studies have been published about the importance of PD-1 and PD-L1 in reproductive immunology. Several investigations, including ours, proved the presence of PD-1 receptors in the surface of decidual immune cells in the 1st trimester of healthy pregnancy [21,22]. Furthermore, other publications reported a sharp PD-L1 expression pattern in the outer side of the syncytiotrophoblast layer but not in the inner side as well as not on cytotrophoblasts [23,24]. Therefore the connection of PD-1 with PD-L1 can be assumed at the materno-fetal region, which could induce immune tolerance mechanisms against the fetus. Besides the membrane-bound receptor-ligand formation, soluble forms of sPD-L1 also can be a part of the PD-1/PD-L1 signaling pathway at the same time; sPD-1 can rather be a competitive inhibitor by blocking this interaction [25]. A recent pregnancy study reported an elevated sPD-L1 level in the serum of healthy pregnant women compared to nonpregnant women [26]. At the same time, another research published a higher sPD-1 level in women with preeclampsia [27]. Further investigations using murine pregnancy models revealed that the blockade of anti-PD-1 or anti-PD-L1 results in insufficient tolerance mechanisms accompanied by the upregulated T-cell response and reduced Treg function at the materno-fetal interface [28]. According to these findings, the PD-1/PD-L1 pathway could have an important role in the establishment of immune tolerance mechanisms at the materno-fetal interface.

NKG2D is an activating receptor present on all murine and human NK cells, which is constitutively expressed by human CD8+T cells and can also be expressed by NKT-like cells, γδ T cells and a small subgroup of CD4+T cells [29]. After binding to its ligands such as UL16 binding proteins (members of the ULBP family), or the MHC-class-related molecules A and B (MICA, MICB) it has a costimulatory function and contribute to the cytotoxic activity of these effector cells [30]. Numerous studies have investigated the importance of NKG2D receptor and ligands in pregnancy-related immunoregulation [31,32,33]. Furthermore, an increasing number of studies have found a connection between the PD-1/PD-L1 immune checkpoint pathway and the NKG2D activatory receptor. In a previous work, we demonstrated a high expression of NKG2D in the surface of decidual PD-1-positive CD8+T cells. We also found that the cytotoxic potential of decidual PD1/NKG2D double-positive CD8+T cells was significantly decreased compared to the peripheral counterparts [21].

Although numerous studies have examined the possible role of the PD-1 and PD-L1 immune checkpoint molecules related to healthy or pathological pregnancies, there is no available data about the expression pattern of these molecules in the periphery during human pregnancy. We aimed to investigate the peripheral PD-1/PD-L1 immune checkpoint pathway and their possible relation with NKG2D throughout the three trimesters of a healthy human pregnancy and in the nonpregnant condition.

## 2. Materials and Methods

### 2.1. Ethical Approval

The study was approved by the local Ethics Committee aligned to the Medical School, University of Pecs (Ethical approval code: 6149-PTE 2019). The study protocol conforms to the ethical guidelines of the 1975 Declaration of Helsinki. Informed written consent was obtained from each participating woman.

### 2.2. Participants and Sample Collection

To investigate the PD-1 and PD-L1 immune checkpoint molecules, 13 healthy women were involved from the first trimester, 10 from the second and 12 from the third trimester of pregnancy in cooperation with Department of Obstetrics and Gynaecology at the University of Pecs (Table 1). Ten nonpregnant, healthy, age-matched women were also recruited to form the control group from the National Blood Bank, Regional Centre, Pecs. Under the EU-GDPR and due to the Privacy, Informational and Health data act, regulations, all personal and health-related data obtained about the donors during blood donation were processed anonymously, confidentially and securely, and were not provided to the research team for further demographic analysis. The health status of the participating women was identified: none of them had a significant medical history, were taking medications or had current or recent illnesses. All women affected by pregnancy-related complications and/or infection, prepregnancy disease, in vitro fertilization pregnancies, immune-associated disease, diabetes mellitus and AIDS were excluded.

Venous blood (10 mL) was taken in heparinized tubes and transported immediately to the laboratory for further investigations.

Statistical comparisons were made in R using one-way ANOVA tests. Data are shown as the mean value (range). Statistical differences among the investigated cohorts were not detected.

### 2.3. Lymphocyte Separation, Cryopreservation and Thawing

Using Ficoll-Paque (GE-Healthcare), density gradient peripheral blood mononuclear cells (PBMC) were isolated from heparinized venous blood. Separated cell fractions were then washed in complete Rosewell Park Memorial Institute medium (RPMI) 1640 supplemented with 10% fetal calf serum (FCS), counted and centrifuged. Cells were resuspended in inactivated human serum containing 10% DMSO for cryoprotection. Next, the cells were aliquoted in cryovials and stored in a −80 °C mechanical freezer. On the day of fluorescent cell labelling, the samples were thawed as quickly as possible in a 37 °C water bath, resuspended in RPMI 1640 medium and washed twice to remove the remaining DMSO content.

### 2.4. Antibodies

For flow cytometric analyses, surface and intracellular staining were performed from freshly thawed PBMC. The following monoclonal antibodies were used in recommended concentration according to the manufacturer: fluorescein isothiocyanate (FITC)-conjugated anti-human CD4 (Clone: RPA-T4, Cat. No.: 555346, BD Biosciences, San Diego, CA, USA), FITC-conjugated anti-human CD107a (Clone H4A3, Cat. No.: 555800, BD Biosciences, San Diego, CA, USA), phycoerythrin (PE)-conjugated anti-human PD-1 (Clone: PD1.3, Cat. No.: B30634, Beckmann-Coulter, Indianapolis, IN, USA), phycoerythrin/Cy7 (PE/Cy7)-conjugated anti-human NKG2D (Clone: 1D11, Cat. No.: 562365, BD Biosciences, San Diego, CA, USA), allophycocyanin (APC)-conjugated anti-human CD56 (Clone: B159, Cat. No.: 555518, BD Biosciences, San Diego, CA, USA), APC-conjugated anti-human FoxP3 (Clone: 236A/E7, Cat. No.: 17-4777-42, eBioscience, Waltham, MA, USA), APC/H7-conjugated anti-human CD8 (Clone: SK1, Cat. No.: 560179, BD Biosciences, San Diego, CA, USA), Brilliant Violet (BV)421-conjugated anti-human PD-L1 (Clone: MIH1, Cat. No.: 563738, BD Biosciences, San Diego, CA, USA). The incubation time of the antibodies was 30 min, except anti-human CD107 (4 h) and anti-human FoxP3 (1 h).

### 2.5. Labelling Lymphocytes for Flow Cytometric Analysis

Briefly, the DMSO-free cell suspensions were incubated with a combination of fluorochrome-conjugated monoclonal antibodies for 30 min in complete darkness. The cells were washed with phosphate-buffered saline (PBS) and fixed in 300 μL PBS containing 1% paraformaldehyde and stored at 4 °C in darkness until fluorescence-activated cell sorting (FACS) analysis. Multicolour flow cytometric measurements were performed by FACS Canto II flow cytometer (BD Biosciences, San Diego, CA, USA) equipped with the FACSDIVA V6. software program (BD Biosciences, San Diego, CA, USA). Data analyses were performed with FCS Express V4 (De Novo Software, Pasadena, CA, USA).

### 2.6. CD107a Cytotoxicity Assay

To evaluate the cytotoxic activity by the CD8+T cells and NKT-like cells, surface expression of CD107a molecule was measured by flow cytometry. After DMSO removal PBMC were incubated in RPMI 1640 medium containing 10% FCS, penicillin and streptomycin (Lonza, Basel, Switzerland), ionomycin (Sigma-Aldrich, Saint Louis, MO, USA) and phorbol myristate acetate (Sigma-Aldrich, Saint Louis, MO, USA) in the presence of anti-human CD107a monoclonal antibody for 4 h at 37 °C in an atmosphere containing 5% CO_2_. At the end of stimulation, the cells were washed in PBS, resuspended, then stained with anti-human CD3 and CD8 or anti-human CD3 and CD56 antibodies together with anti-human PD-1 and anti-human NKG2D antibodies for 30 min at room temperature in complete darkness. Finally, the cells were washed in PBS, fixed with 1% paraformaldehyde (PFA), and analyzed by flow cytometry.

### 2.7. Intracellular Staining of Treg Cells

To examine the Treg cell population, intracellular FoxP3 transcription factor was labelled using the FoxP3 Staining Buffer Set (eBioscience, Waltham, MA, USA) according to the manufacturer protocol. Briefly, after the surface labelling, PBMC were permeabilized in 1 mL fixation/permeabilization buffer (concentrate/diluent 1:4) at 4 °C for 1 h in complete darkness. Next, the samples were washed twice in a staining buffer and stained with the anti-human FoxP3 monoclonal antibody at 4 °C for 1 h in darkness. Then the cells were washed twice again in the staining buffer, fixed with 1% PFA and evaluated by flow cytometry.

### 2.8. Statistical Analysis

Statistical analyses were performed using R, version 3.5.3 [34]. Multiple comparisons were made using one-way ANOVA. PD-1 expression by CD8+ cells, PD-1 expression by NKT-like cells, PD-L1 expression by CD8+ cells, PD-L1 expression by NKT-like cells, percentage of PD-1+ CD8+T in PD-1+ cell population (%), percentage of PD-1+ NKT-like in PD-1+ cell population, percentage of PD-1+/PD-L1 double positive CD8+T cells (%), CD107a expression by PD-1+ CD8+T cells (%), CD107a expression by PD-1- CD8+T cells (%) and CD107a expression by PD-1+ NKT-like T cells (%) were log_e_ transformed after graphical normality testing according to Crawley (2014) [35]. For pairwise comparisons Tukey post hoc tests were applied [36].

To test the relationship between PD1-PDL1 and PD1-NKG2D in each trimester, linear regression analyses were performed. Applicability of linear regression models were tested graphically using QQ-plots and residual plots. *p* values and coefficients of determination (R2) were calculated in R.

### 2.9. Results

Phenotype characteristics of different T cell subpopulations throughout pregnancy and in nonpregnant women.

The ratio of different immune cell subpopulations (CD3+T cells, CD4+T cells, CD8+T cells, Treg cells, and NKT-like cells) was measured using multicolour flow cytometry from the peripheral blood of healthy pregnant and nonpregnant women. Following the comparison of the percentage of the CD3+T, CD4+T, CD8+T, Treg and NKT-like cells, no significant difference was detected between the three trimesters of healthy pregnancy and nonpregnant status (Table 2).

Statistical comparisons were made in R using one-way ANOVA tests. The results were presented as the mean value ± SD. Statistical differences among the investigated cohorts were not detected.

PD-1 receptor and PD-L1 ligand expression by different T cell subpopulations throughout pregnancy and in nonpregnant women.

The surface expression of PD-1 and PD-L1 molecules by CD8+T cells, CD4+T cells, Treg cells and NKT-like cells was measured by multicolour flow cytometry.

**Figure 1:**
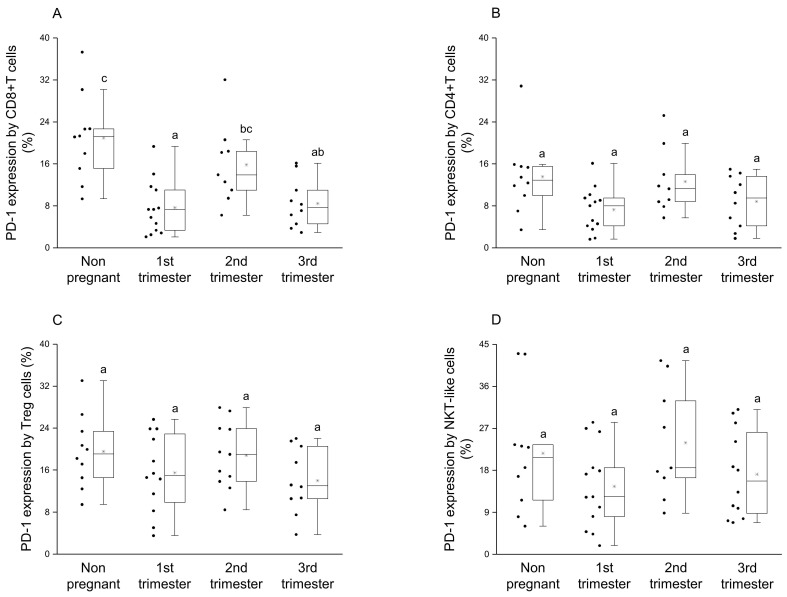
PD-1 receptor expression by different T cell throughout pregnancy and in nonpregnant women. The expression of PD-1 molecule by CD8+T cell (**A**), CD4+T (**B**), Regulatory T (Treg) (**C**) and NKT-like (**D**) subpopulations. The solid bars represent medians of 10, 13, 10 and 12 samples, the boxes indicate the interquartile ranges and the lines show the most extreme observations. Differences were considered statistically significant for *p*-values ≤ 0.05. Variables with the same letter indicate no statistically significant difference, while different letters indicate a statistically significant difference.

Significantly elevated PD-1 expression was measured by the CD8+T cell subset in the nonpregnant cohort compared to the groups from the 1st and the 3rd trimester of pregnancy (Figure 1A). Furthermore, the expression level of the PD-1 receptor by CD8+T cells was significantly higher in 2nd trimester compared to the 1st trimester of pregnancy (Figure 1A). The PD-1 receptor expression by the other investigated subpopulations (CD4+T, Treg and NKT-like) statistically did not differ between pregnant and nonpregnant conditions (Figure 1B–D).

**Figure 2:**
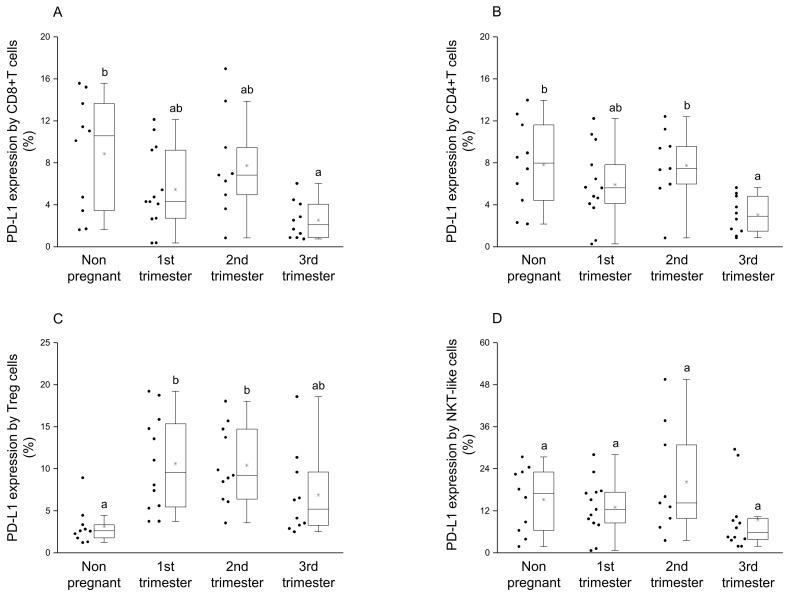
PD-L1 ligand expression by different T cell subpopulations throughout pregnancy and in nonpregnant women. The expression of PD-L1 molecule by CD8+T cell (**A**), CD4+T (**B**), Treg (**C**) and NKT-like (**D**) subpopulations. The solid bars represent medians of 10, 13, 10 and 12 samples, the boxes indicate the interquartile ranges, and the lines show the most extreme observations. Differences were considered statistically significant for *p*-values ≤ 0.05. Variables with the same letter indicate no statistically significant difference, while different letters indicate a statistically significant difference.

By examining the presence of PD-L1 molecule on the surface of CD8+T and CD4+T cells, a significant reduction was observed in the 3rd trimester of pregnancy compared to nonpregnant controls (Figure 2A,B). Moreover, in CD4+T cells significant difference in PD-L1 expression was observed between the 2nd and 3rd trimester of pregnancy (Figure 2B). Analyzing the ligand expression by Treg cells, significantly higher PD-L1 presence was revealed in the 1st and 2nd trimester of pregnancy compared to nonpregnant controls (Figure 2C). In the case of NKT-like cells, a significant difference was not observed among the investigated groups (Figure 2D).

**Figure 3:**
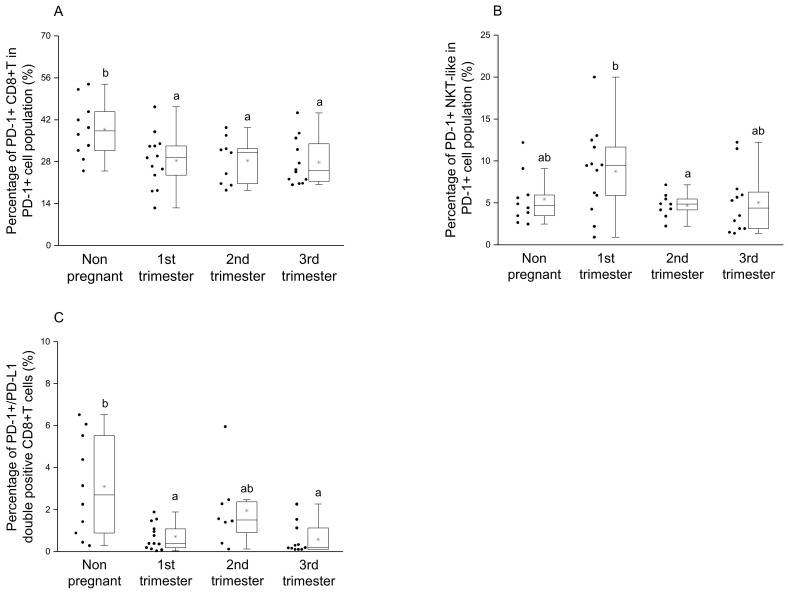
Percentage of PD-1+ immune cell subpopulations in PD-1+ cells and the percentage of PD-1/PD-L1 double-positive CD8+T cells throughout pregnancy and in nonpregnant women. The percentage of the PD-1+ CD8+T cells (**A**) and PD-1+ NKT-like (**B**) cells in the PD-1+ cell population (**A**) and the percentage of PD-1+/PD-L1 double-positive CD8+T cells (**C**) The solid bars represent medians of 10, 13, 10 and 12 samples, the boxes indicate the interquartile ranges, and the lines show the most extreme observations. Differences were considered statistically significant for *p*-values ≤ 0.05. Variables with the same letter indicate no statistically significant difference, while different letters indicate a statistically significant difference.

By investigating the percentage of the PD-1+ CD8+T cells in the PD-1+ cell population, a significant reduction was observed during pregnancy compared to the nonpregnant control group (Figure 3A). Statistical difference in the percentage of the PD-1+ NKT-like cells in PD-1+ cell population was defined only between the 1st and 2nd trimester of pregnancy (Figure 3B). Significantly decreased PD-1/PD-L1 double-positive CD8+T cell subset ratio was also revealed in the 1st and 3rd trimester of pregnancy compared to nonpregnant women (Figure 3C).

Linear regression analyses of PD-1 receptor and PD-L1 ligand molecules on the surface of different T cell subpopulations throughout pregnancy and in nonpregnant women.

**Figure 4:**
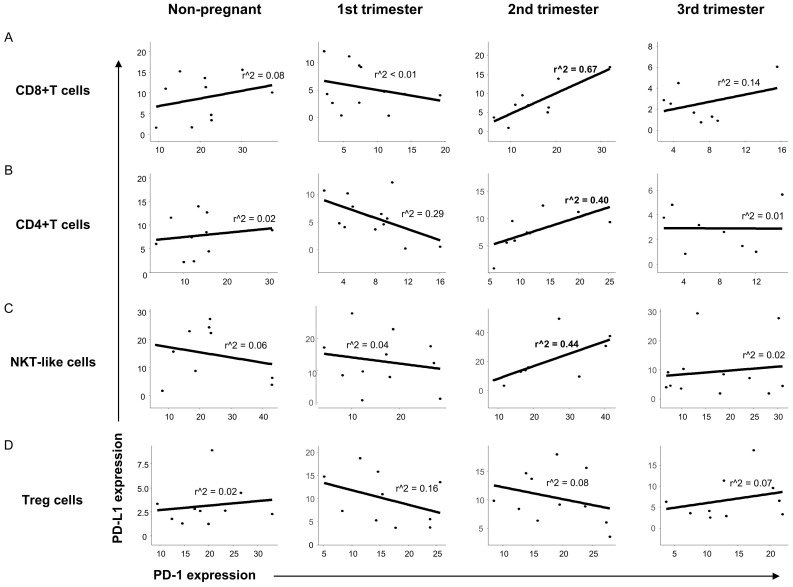
Relationship between the PD-1 receptor and the PD-L1 ligand on the surface of different T cell throughout pregnancy and in nonpregnant women. Linear regression analyses between the expression of PD-1 and PD-L1 surface molecules in CD8+T cells (**A**), CD4+T cells (**B**), NKT-like cells (**C**) and Treg cells (**D**) in women during healthy pregnancy and in nonpregnant women. *p* values and coefficients of determination (R2) were calculated in R.

To reveal a possible relationship between the presence of PD-1 and PD-L1 on the surface of different T cell subsets, linear regression analyses were performed. In the case of CD8+T, CD4+T and NKT-like cells a positive relationship between the expression of PD-1 and PD-L1 was observed only in the 2nd trimester of pregnancy (Figure 4A–C) at the same time, we did not find any relationship in the case of the Treg cell population (Figure 4D).

Connection between NKG2D activator receptor and PD-1 inhibitory immune checkpoint receptor expression on CD8+T cells throughout pregnancy and in nonpregnant women.

**Figure 5:**
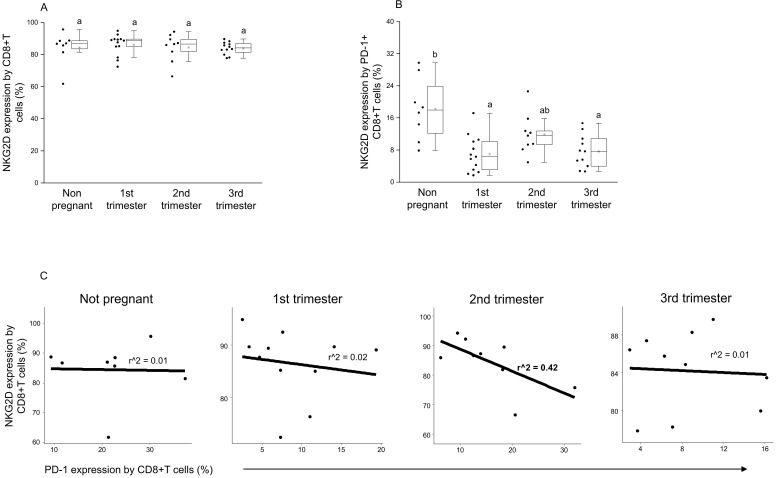
Connection between NKG2D activator receptor and PD-1 inhibitory immune checkpoint receptor expression on CD8+T cells throughout pregnancy and in nonpregnant women. The surface expression of NKG2D receptor by CD8+T cells (**A**) and PD-1+ CD8+T cells (**B**). The solid bars represent medians of 10, 13, 10 and 12 samples, the boxes indicate the interquartile ranges and the lines show the most extreme observations. Differences were considered statistically significant for *p*-values ≤ 0.05, Variables with the same letter indicate no statistically significant difference, while different letters indicate a statistically significant difference. Linear regression analyses between PD-1 and NKG2D surface receptor expressions by CD8+T cells (**C**) in women during healthy pregnancy and in nonpregnant women. *p* values and coefficients of determination (R2) were calculated in R.

By examining the NKG2D expression by the CD8+T cell population, no significant difference was detected between the investigated groups (Figure 5A). At the same time, significantly decreased activator receptor expression was measured by the PD-1+ CD8+T cells in the 1st and 3rd trimester of pregnancy compared to nonpregnant controls (Figure 5B). Furthermore, after a linear regression test, a negative relationship was observed between NKG2D and PD-1 receptor expression on the surface of CD8+T cells in the 2nd trimester of pregnancy only (Figure 5C).

Cytotoxic potential of peripheral PD-1 positive and negative CD8+T cells and NKT-like cells during pregnancy and in nonpregnant women.

**Figure 6:**
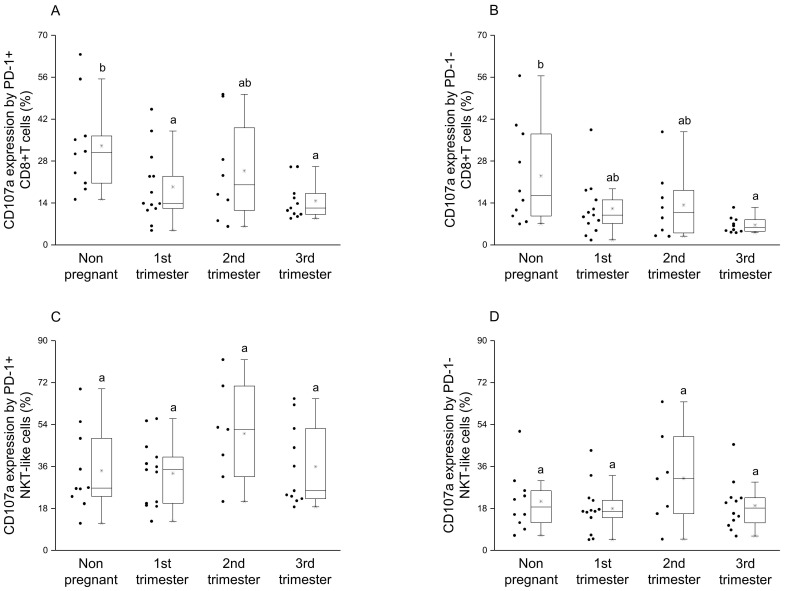
Cytotoxic activity of peripheral PD-1 positive and negative CD8+T cells and NKT-like cells throughout pregnancy and in nonpregnant women. The expression of CD107a molecule by PD-1 positive (**A**), PD-1 negative (**B**) CD8+T cells and PD-1 positive (**C**), PD-1 negative (**D**) NKT-like cells. The solid bars represent medians of 10, 13, 10 and 12 samples, respectively, the boxes indicate the interquartile ranges and the lines show the most extreme observations. Differences were considered statistically significant for *p*-values ≤ 0.05. Variables with the same letter indicate no statistically significant difference, while different letters indicate a statistically significant difference.

By examining the CD107a expression by the PD-1 positive CD8+T cells, a significant decrease was observed in the 1st and 3rd trimester of pregnancy compared to the nonpregnant condition (Figure 6A), at the same time, a significant difference was detected in degranulation by the PD-1 negative CD8+T cells between the 3rd trimester and the nonpregnant group (Figure 6B). In the case of NKT-like cells, a statistical difference was not revealed in cytotoxicity by the PD-1 positive and negative subsets among the investigated groups (Figure 6C,D).

Linear regression of CD107a expression by PD-1 and NKG2D positive/negative subpopulations in CD8+T and NKT-like cells.

Analyzing the possible relationship between NKG2D activatory and PD-1 inhibitory receptors, a linear regression of the cytotoxic activity was measured by receptor-positive and negative CD8+T and NKT-like cells subpopulations.

**Figure 7:**
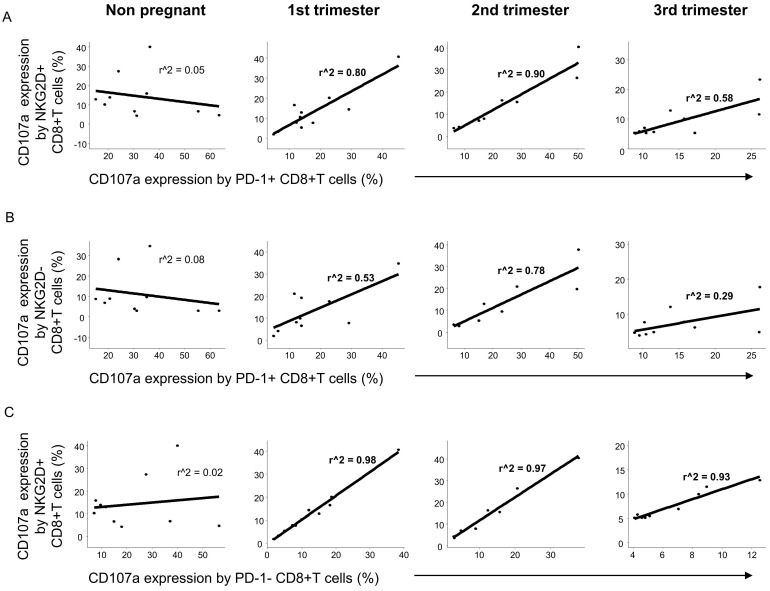
The regression of the CD107a expression by PD-1 and NKG2D positive/negative subpopulations in CD8+T cells throughout pregnancy and in nonpregnant women. Linear regression analyses between the CD107a expression by the PD-1+/NKG2D+ (**A**), PD-1+/NKG2D− (**B**) and PD-1−/NKG2D+ (**C**) subpopulations in CD8+T cells in women during healthy pregnancy and in nonpregnant women. *p* values and coefficients of determination (R2) were calculated in R.

Positive linear regression was observed between the cytotoxic activity of PD-1 positive and NKG2D positive CD8+T cells during the three trimesters of pregnancy but not in nonpregnant conditions (Figure 7A). A weaker relationship was detected in the cytotoxicity of PD-1 positive and NKG2D negative CD8+T cells only in the 1st and 2nd trimester of pregnancy (Figure 7B), while in the case of the CD107a expression by the PD-1 negative and NKG2D positive CD8+T subsets a stronger linear regression was determined during pregnancy but not in nonpregnant women (Figure 7C).

**Figure 8:**
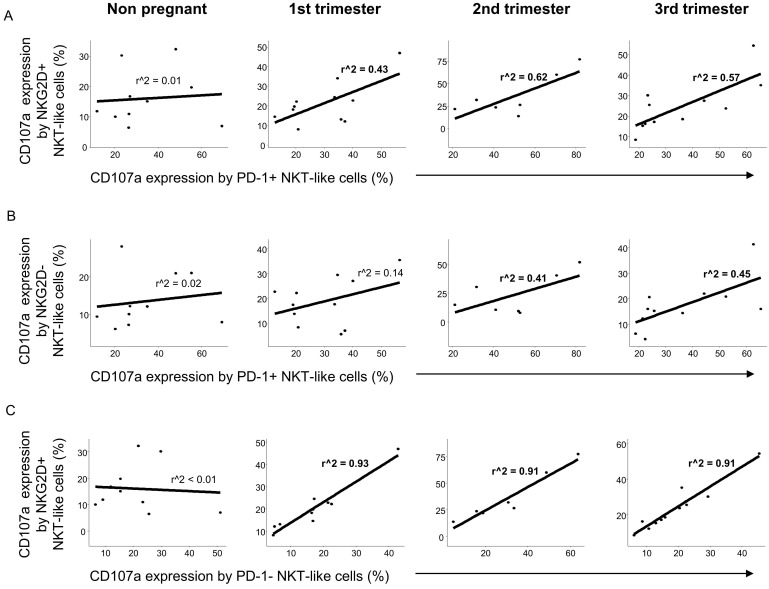
Regression analyses of the CD107a expression by PD-1 and NKG2D positive/negative subpopulations in NKT-like cells throughout pregnancy and in nonpregnant women. Linear regression analyses between the CD107a expression by the PD-1+/NKG2D+ (**A**), PD-1+/NKG2D− (**B**) and PD-1−/NKG2D+ (**C**) subpopulations in NKT-like cells in women during healthy pregnancy and in nonpregnant women. *p* values and coefficients of determination (R2) were calculated in R.

Similarly to CD8+T cells, a positive relationship was observed between the cytotoxic activity of the PD-1 positive and NKG2D positive NKT-like cell populations throughout pregnancy but not in the nonpregnant control group (Figure 8A). Examining the connection of the CD107a expression by PD-1 positive and NKG2D negative NKT-like cells, a weak positive relationship was observed only in the 2nd and 3rd trimester of pregnancy (Figure 8B). Moreover, strong positive linear regression was defined between the cytotoxicity in PD-1 negative and NKG2D positive NKT-like cells during the healthy pregnancy but not nonpregnant condition (Figure 8C).

## 3. Discussion

Immune checkpoint molecules are primarily investigated in tumor immunology, although notable literature exists about their importance in other fields of immunology like autoimmunity, transplant immunology or infection immunology. Although the knowledge about the immune checkpoint pathways in reproductive immunology is emerging, and mainly focusing on the materno-fetal interface, the exact role of these regulator molecules is still unknown, especially in the periphery. Our previous work is the only one to examine immune checkpoint molecules from peripheral blood during the three trimesters of a healthy pregnancy, revealing notable differences regarding the TIM-3/Gal-9 checkpoint inhibitor pathway, especially in NK cells [37]. Since only CD56dim NK cells express a high level of PD-1 receptor in 25% of healthy individuals, in this study, we focused only on different T cell populations, their PD-1/PD-L1 expression and its connection with the activatory receptor NKG2D.

After phenotypical analyses of PD-1 receptor, a significant difference was detected only in the case of CD8+T cells, which shows the importance of the inhibitory effect on cytotoxic T cells. A significant decrease regarding the expression of inhibitory PD-1 receptor in the 1st and 3rd trimester of pregnancy could be a part of the above-mentioned Th1 predominance in these trimesters. We hypothesize that the Th1 predominance could lead to the increase of PD-1 expression by CD8+T cells to a level seen in the 2nd trimester. For the implementation of the PD-1 mediated inhibitory effect, the presence of the ligand molecule is also crucial. We found a decrease of PD-L1 expression on the surface of CD4+T and CD8+T cells in the 3rd trimester of pregnancy, which could affect the PD-1/PD-L1 pathway resulting in a Th1 predominance before and during delivery. Although, a recent publication revealed an increased soluble PD-L1 level in women from the 3rd trimester of pregnancy [38], which seems contradictory to our results. The source of circulating PD-L1 molecules could be either on placental trophoblast or from the surface of PD-L1 positive CD4+T and CD8+T cells, since it has not been defined in pregnancy yet [26,27,39,40]. The regulatory role of Treg cells during pregnancy is extensively investigated [41,42,43] hence the increased level of the PD-L1 molecule on the surface of Tregs in the 1st trimester of pregnancy in parallel with the elevated PD-L1 expression by decidual Treg cells [21,44], which could be part of the controlled inflammation process during implantation and early pregnancy. At the same time, the higher expression level of PD-L1 molecule by Treg cells in the 2nd trimester of pregnancy may help to maintain Th2 predominance by inhibiting the effector Th1 cells through binding to PD-1. In connection with these findings, an interesting relationship was revealed in the expression level of PD-1 and PD-L1 by the same cell surface only in the 2nd trimester of pregnancy. A recently published study considers a possible option that PD-L1/PD-1 cis interaction on the surface of T cells could trigger a signaling pathway in the absence of trans ligands or receptors [45]. A positive regression between PD-1 and PD-L1 could be a self-regulating mechanism by the surface of CD8+T, CD4+T and NKT-like cells, and may contribute to the Th2 character in the 2nd trimester.

To clarify the possible role of the PD-1/PD-L1 immune checkpoint pathway, a novel activatory receptor NKG2D was involved in our investigations, which is highly expressed on the surface of CD8+T and NKT-like cells and stable during pregnancy. A recent study published interesting data that a high level of circulating NKG2D ligands are associated with poor efficacy of PD-1/PD-L1 immune checkpoint therapy [46]. Hence we tested the connection between PD-1 and NKG2D receptors on the surface of CD8+T and NKT-like cells. Investigating the NKG2D receptor expression by CD8+T cells did not find any significant difference between the investigated groups. However a negative relationship was revealed between NKG2D and PD-1 receptors regarding CD8+T cells in the 2nd trimester of pregnancy. The increased PD-1 expression by the same CD8+T cell subset might relate to the Th2 predominance in the 2nd trimester of pregnancy.

After phenotypical examinations, CD107a degranulation assay was performed to collect further information about the functionality of PD-1 and NKG2D expressing CD8+T and NKT-like cells. A significantly decreased CD107a expression only by the PD-1 expressing CD8+T cells in 1st trimester of pregnancy may be a part of a compensatory mechanism via downregulating cytotoxic T cells maintaining the immunological balance in this Th1 mediated trimester. This study also found that PD-1-positive CD8+T cells showed a significant decrease in CD107a expression in the 1st and 3rd trimester of pregnancy compared to the nonpregnant condition while PD-1-negative CD8+T cells had a significantly decreased degranulation only in the 3rd trimester compared to the nonpregnant group. The decreased cytotoxic potential regarding PD-1-positive CD8+T cells could be partly explained by their exhaustion. A strong positive relationship in the cytotoxicity of PD-1- and NKG2D-positive CD8+T cells during pregnancy may indicate the importance of the CD107a degranulation in maternal immune regulation, although it seems independent from PD-1 or NKG2D expression in the periphery. Nevertheless, cytotoxic activity showed a really strong regression between the PD-1-negative and NKG2D-positive CD8+T cells, which might relate to maternal immune responses toward the presence of paternal antigens.

In summary, our results about the peripheral expression of PD-1, PD-L1 and the connection with NKG2D, primarily in the case of CD8+T and NKT-like cells, could contribute to the changes of the Th1/Th2/Th1 predominance and can help to maintain maternal immunotolerance in the periphery too.

## Figures and Tables

**Table 1:** Gynecological and demographic data of the participating women.

	Nonpregnant	1st Trimester	2nd Trimester	3rd Trimester
No. of women	10	13	10	12
Age (years)	31 (19–44)	29 (20–34)	30 (26–35)	33.5 (26–43)
Gestation age at sampling (weeks)	-	12.09 (11–14)	26.18 (25–28)	36.00 (35–37)
Gestation age at birth (weeks)	-	37.55 (36–41)	39.11 (38–41)	38.80 (37–41)
Gravidity	-	2.70	1.91	3.00
Parity	-	1.60	1.00	1.00

**Table 2:** Phenotype characteristics of peripheral blood mononuclear cell populations throughout pregnancy and in nonpregnant women.

	Nonpregnant	1st Trimester	2nd Trimester	3rd Trimester
CD3+T cells	63.06 ± 8.65	67.36 ± 6.81	66.14 ± 7.66	70.77 ± 9.68
CD8+T cells	20.91 ± 5.42	23.18 ± 5.77	18.51 ± 3.66	23.21 ± 8.38
CD4+T cells	36.07 ± 8.38	35.50 ± 7.89	40.41 ± 8.73	42.15 ± 9.67
Treg cells	2.19 ± 0.77	1.71 ± 0.79	2.75 ± 1.56	2.66 ± 1.38
NKT-like cells	3.55 ± 3.37	3.74 ± 2.15	2.34 ± 1.06	2.52 ± 2.01
